# Autoimmune Skin Disease Exacerbations Following COVID-19 Vaccination

**DOI:** 10.3389/fimmu.2022.899526

**Published:** 2022-05-27

**Authors:** Grant Sprow, Mohsen Afarideh, Joshua Dan, Rui Feng, Emily Keyes, Madison Grinnell, Josef Concha, Victoria P. Werth

**Affiliations:** ^1^ Dermatology, Corporal Michael J. Crescenz Department of Veterans Affairs Medical Center, Philadelphia, PA, United States; ^2^ Dermatology, Perelman School of Medicine, University of Pennsylvania, Philadelphia, PA, United States; ^3^ Department of Biostatistics, Epidemiology, and Informatics, University of Pennsylvania, Philadelphia, PA, United States

**Keywords:** vaccination, COVID-19, autoimmune, connective tissue disease, skin

## Abstract

**Background:**

Vaccination against COVID-19 reduces the risk of severe COVID-19 disease and death. However, few studies have examined the safety of the COVID-19 vaccine in patients with autoimmune skin disease.

**Objectives:**

We sought to determine the incidence of disease exacerbation in this population following COVID-19 vaccination as well as the associated factors.

**Methods:**

We performed a chart review of all patients seen in the autoimmune skin disease clinic of the principal investigator during the study period. All patients included for analysis were systematically and prospectively asked about COVID-19 vaccination status, manufacturers, vaccine dates, autoimmune symptoms after the vaccine, and timing of symptom onset using a standardized template as part of their visit. Demographics and autoimmune disease diagnosis were also collected. Analysis used Chi-square and Fisher’s exact tests.

**Results:**

402 subjects were included for analysis. 85.6% of patients were fully vaccinated, with 12.9% unvaccinated and 1.5% partially vaccinated. 14.8% of fully vaccinated patients reported worsening autoimmune signs and symptoms after the vaccine. Fully vaccinated dermatomyositis patients were more likely to report worsening autoimmune signs and symptoms after the vaccine (22.7%) than fully vaccinated lupus erythematosus patients (8.6%) (p=0.009). Patients fully vaccinated with the Moderna vaccine trended towards an increased likelihood of reporting worsening autoimmune signs and symptoms after the vaccine (19.1%) than those with the Pfizer-BioNTech vaccine (12.0%) (p=0.076). Of the patients who had autoimmune symptoms after vaccination, 20% had symptoms after the 1st dose, 82% after the 2nd dose, and 4% after the 3rd dose with median onset (95% confidence interval) of 7 (2,14), 14 (14,21), and 18 (7,28) days later, respectively.

**Conclusions:**

More fully vaccinated dermatomyositis patients had exacerbation of autoimmune signs and symptoms after the vaccine than fully vaccinated lupus erythematosus patients. However, given the risks of COVID-19, clinicians should still promote vaccination in most patients with autoimmune skin disease.

## Introduction

COVID-19, the disease resulting from infection with the SARS-CoV-2 virus, was officially declared a pandemic by the World Health Organization on March 11, 2020. Since then, COVID-19 has led to enormous amounts of death, altered how society approaches many aspects of daily life, and placed significant strain on healthcare systems worldwide (1). Development of vaccines targeting SARS-CoV-2 occurred at a historically quick pace and led to FDA emergency use authorization of two vaccines using mRNA technology administered in a two-dose series (Pfizer-BioNTech and Moderna) and one adenovirus-based vaccine administered in one dose (Johnson & Johnson). The Pfizer-BioNTech and Moderna vaccines eventually received full FDA approval, and booster doses became recommended 5 months after the initial two-dose vaccine series or 2 months after a Johnson & Johnson (J&J) vaccine.

All of the COVID-19 vaccines used in the United States have been shown to significantly reduce the risk of hospitalization and death (2). However, many patients with autoimmune diseases experience hesitancy with regards to receiving a COVID-19 vaccine (3). Patients with immune-mediated inflammatory diseases were not included in the COVID-19 vaccine clinical trials, leading to questions about vaccine efficacy and safety in this patient population (4, 5). A few studies have examined the incidence of flares in rheumatic disease patients following COVID-19 vaccination and found a range of 3%-13.4% (6–8). However, these studies used either online patient surveys or physician-reported registries to generate their data, which could be possible limitations in determining true flare incidence rates.

The exact mechanisms by which the COVID-19 vaccines could trigger autoimmune disease flares have not been fully elucidated, but some studies may give potential clues. New onset blistering diseases, including bullous pemphigoid (BP), have been reported following the COVID-19 vaccine (9, 10). One study showed that lesions in patients with new onset BP after COVID-19 vaccination had a high clonality of T cells reactive to SARS-CoV-2 derived epitopes while control tissues from prior to the widespread use of COVID-19 vaccines did not (10). This indicates that in these patients there was an ongoing adaptive immune response likely resulting from the vaccine and driving BP lesion formation (10). Thus, the COVID-19 vaccine may lead to an unintended T cell response that can result in new onset autoimmune disease or possibly drive flares in patients who already have an autoimmune disease diagnosis.

Given the lack of knowledge of the safety of the COVID-19 vaccine in patients with autoimmune disease, the aim of our study was to determine the incidence of disease exacerbation in patients with autoimmune skin disease following COVID-19 vaccination as well as to assess the factors associated with these flares.

## Methods

### Study Design

All patients included for analysis were systematically and prospectively asked questions by the principal investigator (PI), author VPW, using a standardized template as part of their standard of care visit as shown here:

Did you get the vaccine? ***
When? ***
If yes, did you have a reaction to the vaccine? ***
When? ***
Which vaccine did you get? ***

We performed a chart review in Epic of all patients seen in the autoimmune skin disease clinic of the PI at the University of Pennsylvania during the study period to collect this prospectively collected data. Data was manually extracted and stored in a password-protected Excel file. Data collected included demographic data, autoimmune disease diagnosis, and data about the COVID-19 vaccine and reactions obtained from the template. Characteristics of the study population are summarized in **Table 1**. Patients were considered unvaccinated if they had never received a COVID-19 vaccine, partially vaccinated if they received only one dose of the Pfizer-BioNTech or Moderna vaccines, and fully vaccinated if they received at least one dose of the J&J vaccine or at least two doses of any vaccines. Patients were categorized into the following autoimmune disease diagnoses based on chart review: lupus erythematosus (LE), dermatomyositis (DM), morphea, pemphigus, bullous pemphigoid (BP), Behcet’s disease, mucous membrane pemphigoid (MMP), and other. The pemphigus group included patients with pemphigus vulgaris and pemphigus foliaceus. The “other” group of autoimmune disease diagnoses included patients who had multiple overlapping autoimmune disease diagnoses as well as others not included here with only 1-2 patients per diagnosis. A full list of autoimmune disease diagnoses included in the “other” group can be found in **Table 2**. Responses recorded in the template were assessed to determine if the patient experienced an autoimmune disease exacerbation (e.g., increased erythema and itchiness of dermatomyositis skin lesions or increased swelling of joints in patients with lupus arthritis) with careful attention to distinguish from typical known vaccine-related adverse events (such as fever and sore arm). This assessment of whether or not patients experienced autoimmune disease exacerbations after receiving the COVID-19 vaccine was also collected. Patients’ interim or initial history, physical exam findings, and assessment and plan were also collected and were assessed to determine if any patients who experienced autoimmune disease exacerbations after the vaccine required an escalation in treatment. Patients who experienced autoimmune disease exacerbations were called to ask if they had stopped any of their autoimmune disease treatments at the time of vaccination, as some patients stopped their immunosuppressive medications in an attempt to increase vaccine response. This study was approved by the institutional review board at the University of Pennsylvania and was carried out in accordance with the Declaration of Helsinki and the Health Insurance Portability and Accountability Act.

**Table 1:** Characteristics of the study population.

Characteristic	Patients (N=402) *No.* (%)
**Age Group**	
** <18**	1 (0.2)
** 18-30**	28 (7.0)
** 31-40**	35 (8.7)
** 41-50**	63 (15.7)
** 51-60**	102 (25.4)
** 61-70**	103 (25.6)
** 71-80**	52 (12.9)
** 80+**	18 (4.5)
**Sex**	
** Male**	74 (18.4)
** Female**	328 (81.6)
**Race**	
** White**	262 (65.2)
** Black**	71 (17.7)
** Asian/Pacific Islander**	25 (6.2)
** American Indian/Alaskan Native**	1 (0.2)
** Other**	16 (4.0)
** Unknown**	27 (6.7)
**Ethnicity**	
** Non-Hispanic**	382 (95.0)
** Hispanic**	7 (1.7)
** Unknown**	13 (3.2)
**Autoimmune Disease Diagnosis**	
** Lupus Erythematosus**	105 (26.1)
** Dermatomyositis**	133 (33.1)
** Morphea**	13 (3.2)
** Pemphigus**	19 (4.7)
** Bullous Pemphigoid**	13 (3.2)
** Behcet’s Disease**	2 (0.5)
** Mucous Membrane Pemphigoid**	10 (2.5)
** Other**	107 (26.6)
**COVID-19 Vaccination Status**	
** Unvaccinated**	52 (12.9)
** Fully Vaccinated**	344 (85.6)
** Partially Vaccinated**	6 (1.5)
**COVID-19 Vaccine Manufacturer**	First dose, second dose, third dose
** Pfizer-BioNTech**	187 (46.5), 184 (45.8), 25 (6.2)
** Moderna**	143 (35.6), 140 (34.8), 14 (3.5)
** J&J**	19 (4.7), 0 (0.0), 1 (0.2)
** Unknown**	1 (0.2), 1 (0.2), 0 (0.0)
** n/a**	52 (12.9), 77 (19.2), 362 (90.0)

**Table 2:** Autoimmune diseases included as “other”.

Other Autoimmune Diseases	
Alopecia areata	Mixed connective tissue disease
CREST syndrome	Multicentric reticulohistiocytosis
Cutaneous polyarteritis nodosa	Neutrophilic dermatosis
Cutaneous sarcoidosis	Neutrophilic urticarial dermatosis
Diabetic dermopathy	Overlap of multiple autoimmune diseases
Disseminated porokeratosis	PASH syndrome
Eczema	Primary Sjogren syndrome
Eczematous dermatitis	Psoriasis
Epidermolysis bullosa acquisita	Pyoderma gangrenosum
Erythema nodosum	Raynaud’s phenomenon
Erythromelalgia	Rheumatoid nodules
Frontal fibrosing alopecia	Sneddon Wilkinson disease
Granuloma annulare	Solar urticaria
Hypocomplementemic urticarial vasculitis	Sweet’s syndrome
IgA vasculitis	Systemic sclerosis
Lichen planopilaris	Thyroid acropachy
Lichen planus	Unclear blistering disease
Lichen sclerosis	Undifferentiated connective tissue disease
Lichenoid mucositis	Urticarial eruption
Livedo vasculopathy	Urticarial hypersensitivity reaction
Livedoid vasculitis	Vitiligo

### Statistical Analysis

Descriptive statistics were performed on patient demographic data, autoimmune disease diagnosis, vaccine manufacturer, and timing of autoimmune disease symptom onset after vaccination. Further analyses used Chi-square and Fisher’s exact tests. Analysis was conducted with IBM SPSS Statistics for Windows, Version 26.0 (Armonk, NY).

## Results

402 subjects were included for analysis. Ages ranged from 17-96 with a median age (95% confidence interval) of 58 (56,60). Most patients (81.6%) were female. Patients came from a variety of racial backgrounds, with 65.2% white and 17.7% Black. The study population was largely non-Hispanic (95.0%). Most patients (59.2%) had either LE or DM. Patients were largely fully vaccinated (85.6%). More patients received the Pfizer-BioNTech vaccine for all 3 doses than other vaccine manufacturers while J&J was given to the least number of patients.

**Figure 1:**
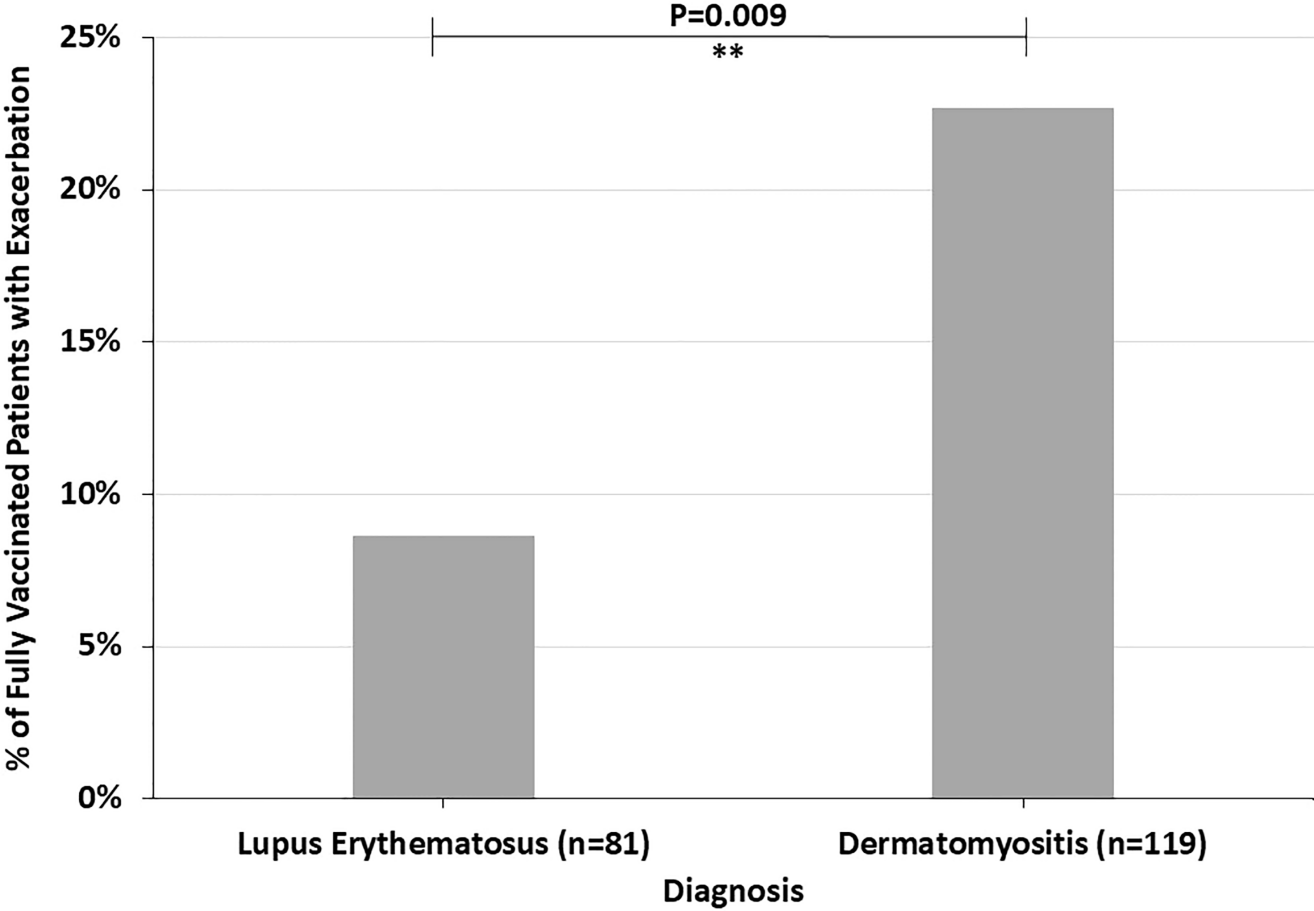
The percent of fully vaccinated patients reporting symptoms of autoimmune disease exacerbation after the COVID-19 vaccine by diagnosis, highlighting the higher incidence in dermatomyositis patients compared to lupus erythematosus patients. **P ≤ 0.01.

**Figure 2:**
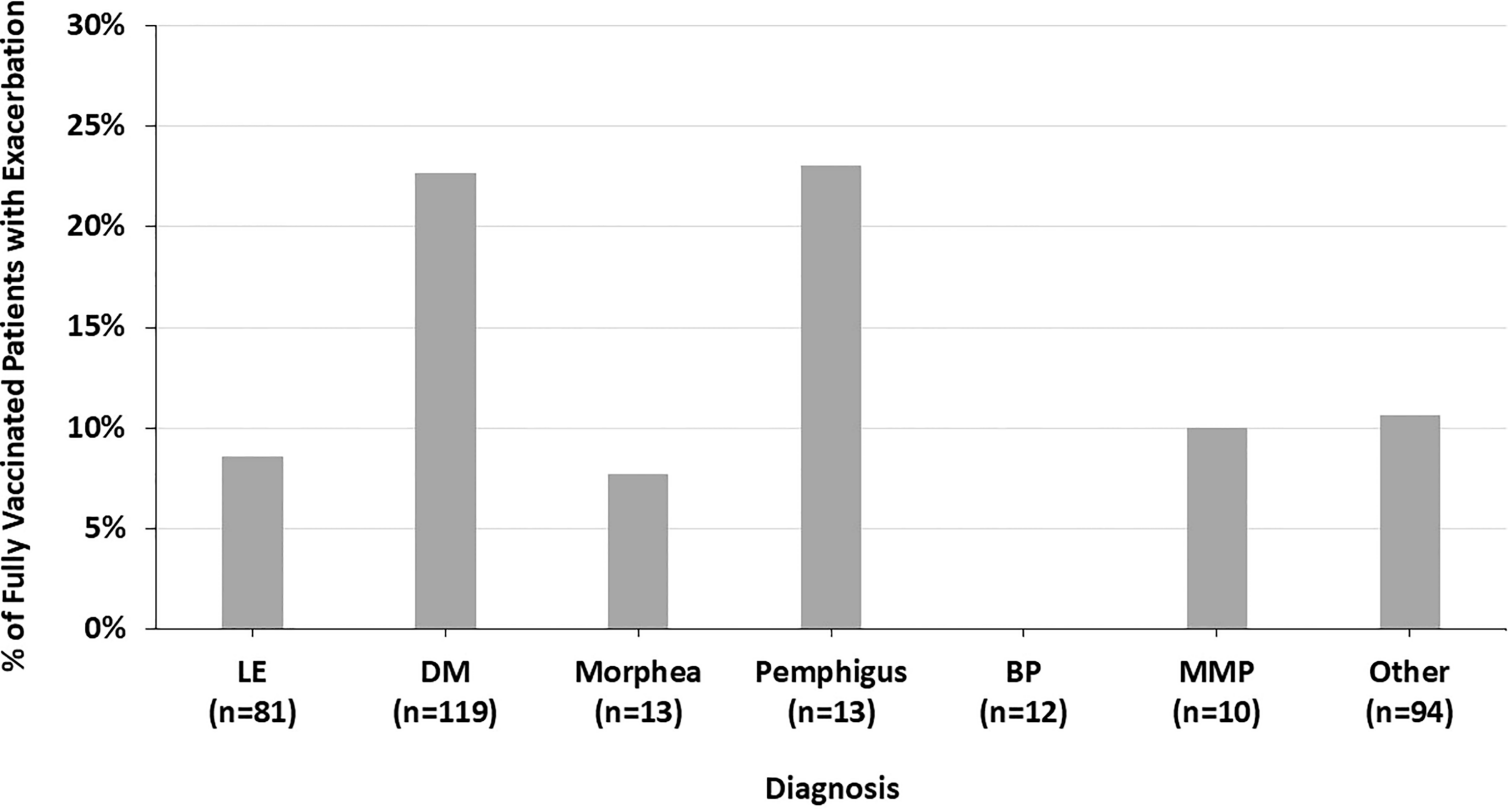
The percent of fully vaccinated patients reporting symptoms of autoimmune disease exacerbation after the COVID-19 vaccine by diagnosis, highlighting the variation across all diagnoses seen.

Across all diagnoses, 14.8% of fully vaccinated patients had autoimmune disease exacerbations or flares after the COVID-19 vaccine. 45.1% of these patients required an escalation in treatment, representing 6.7% of all fully vaccinated patients. 19.6% of fully vaccinated patients who experienced flares had stopped their autoimmune disease treatment at the time of their COVID-19 vaccine. Most of these patients paused their autoimmune disease medications for 1-2 weeks starting at the time of receiving a vaccine dose. Fully vaccinated DM patients were more likely to report an autoimmune disease exacerbation after the COVID-19 vaccine (22.7%) than fully vaccinated LE patients (8.6%) (p=0.009) (**Figure 1**). The two fully vaccinated Behcet’s disease patients both experienced significant flares after the COVID-19 vaccine. These patients were not included in the “other” category to draw attention to the severity of their flares. The proportion of fully vaccinated patients who had autoimmune disease exacerbations after COVID-19 vaccination varied by diagnosis (p=0.004) (**Figure 2**).

**Figure 3:**
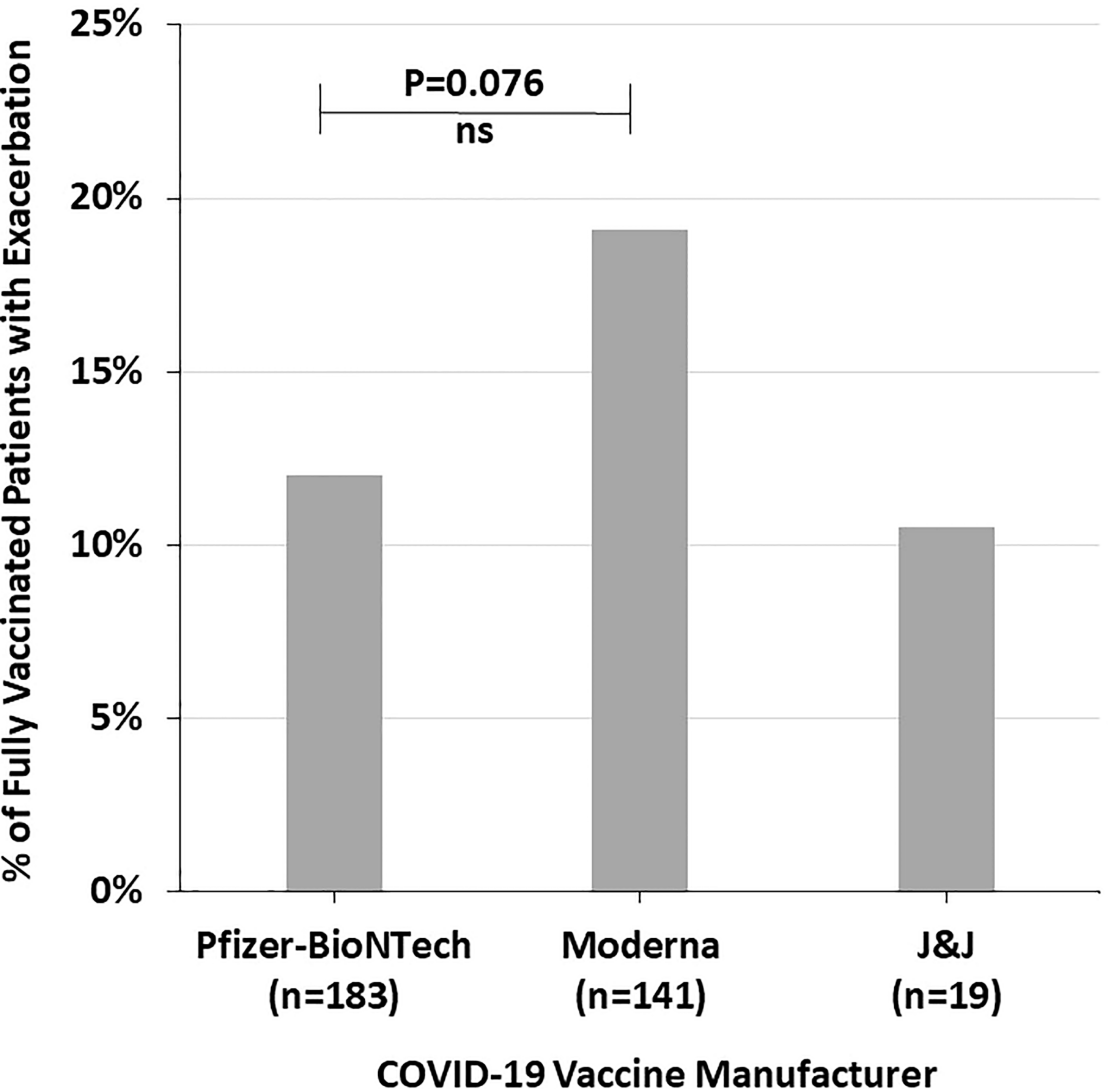
The percent of fully vaccinated patients reporting symptoms of autoimmune disease exacerbation after the COVID-19 vaccine by vaccine manufacturer, highlighting the trend of increased incidence with the Moderna vaccine. NS, Not significant.

**Figure 3** shows the proportion of fully vaccinated patients who reported autoimmune disease exacerbation by COVID-19 vaccine manufacturer. There was a trend towards more fully vaccinated patients who received the Moderna vaccine having an autoimmune disease exacerbation (19.1%) compared to Pfizer-BioNTech (12.0%) and J&J (10.5%) (p=0.198) (**Figure 3**).

**Figure 4:**
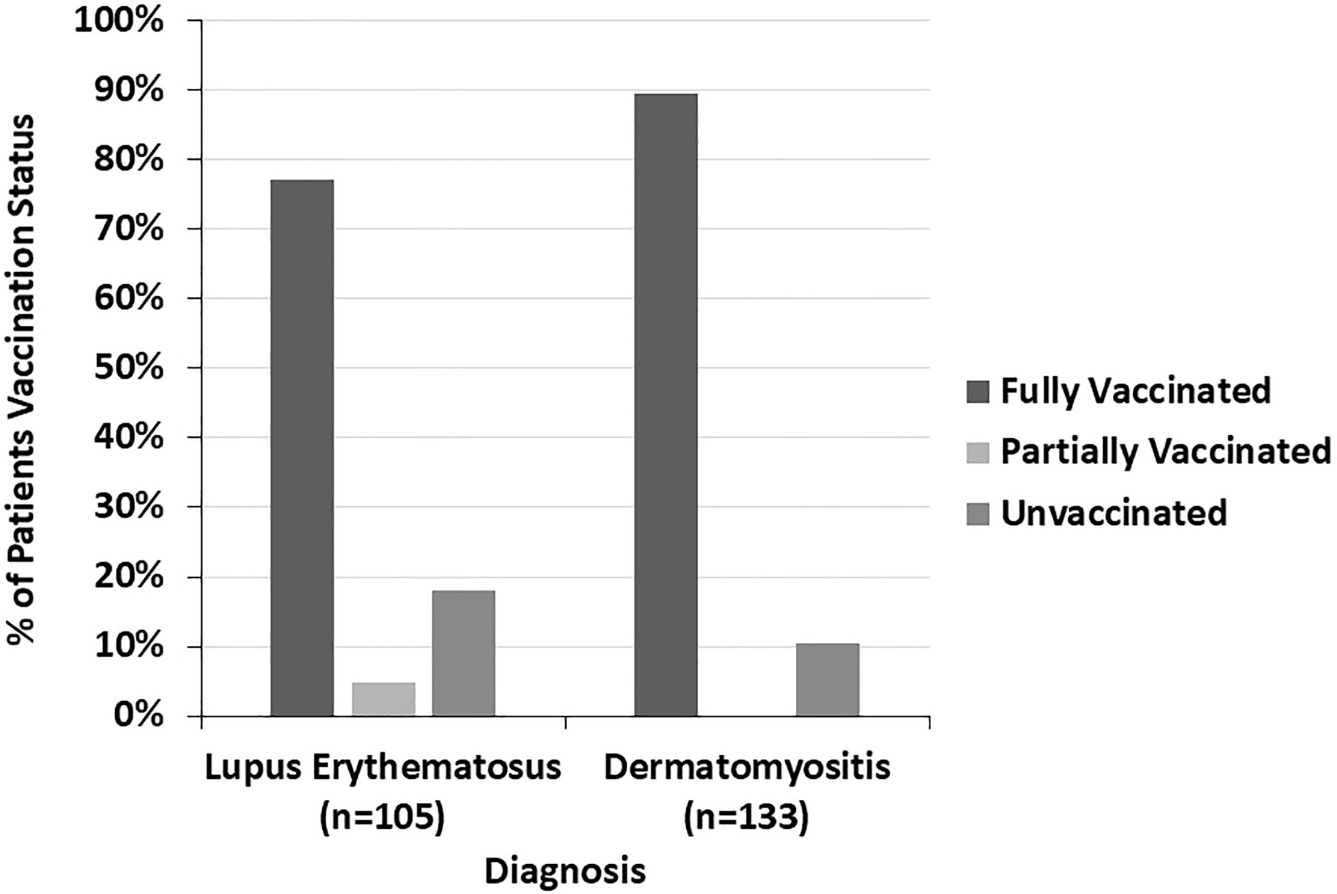
The COVID-19 vaccination status of lupus erythematosus and dermatomyositis patients is shown here. A higher proportion of dermatomyositis patients were fully vaccinated compared to lupus erythematosus patients.

**Figure 5:**
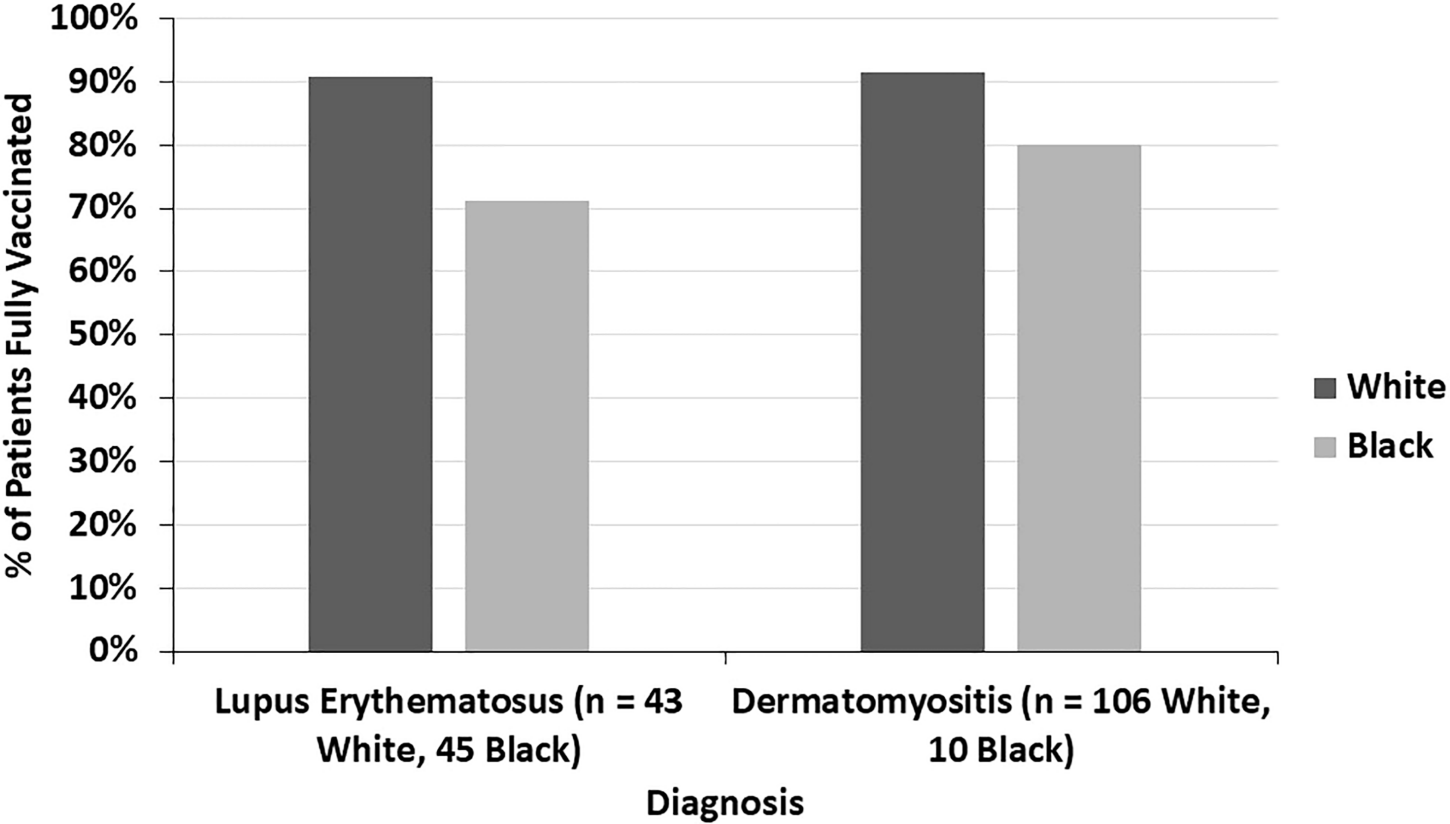
The proportion of fully vaccinated lupus erythematosus and dermatomyositis patients is seen here by racial background (white and Black). In both diseases, a higher proportion of white patients were fully vaccinated compared to Black patients.

**Figure 6:**
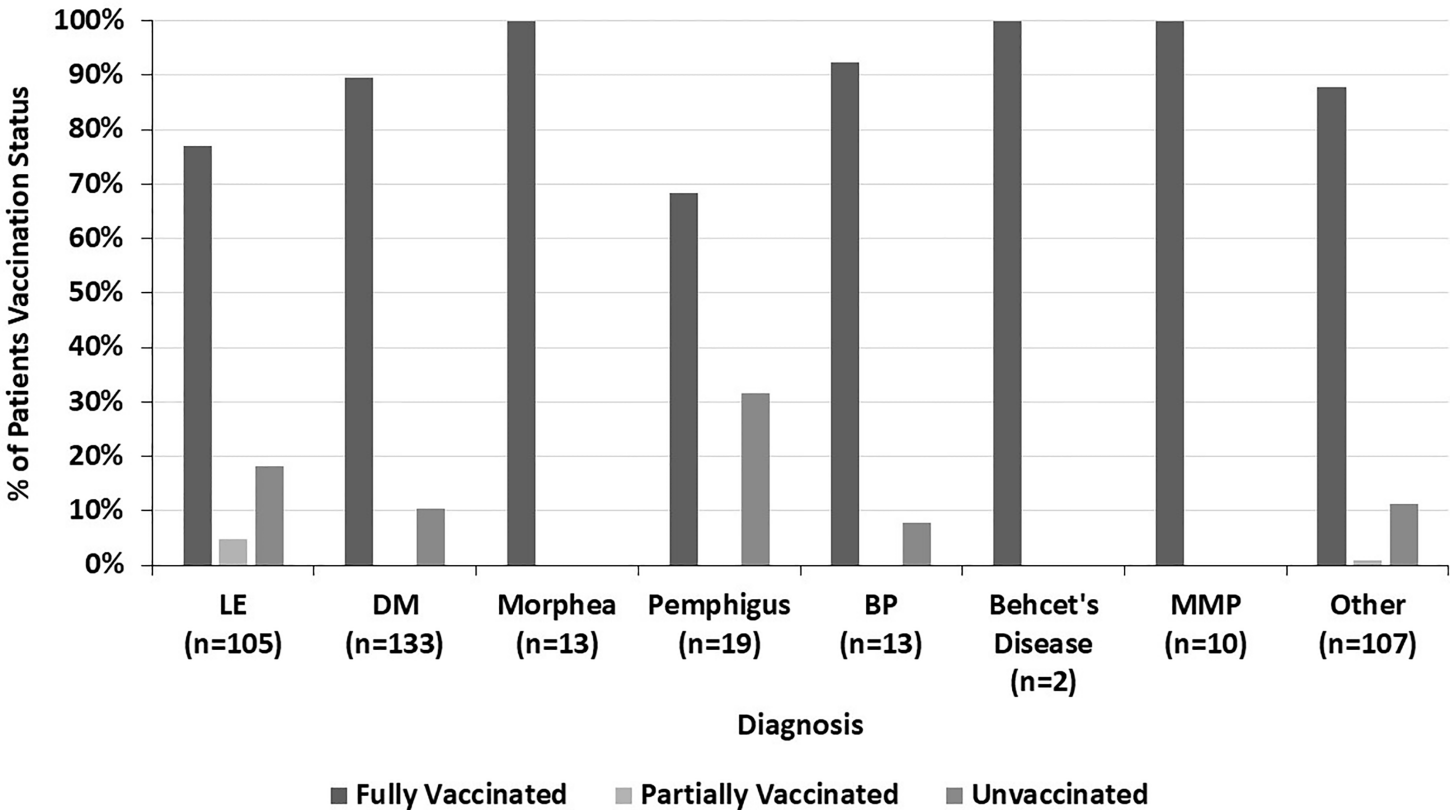
The percent of patients who are fully vaccinated, partially vaccinated, and unvaccinated within each diagnosis, highlighting the variation across all diagnoses seen.

The COVID-19 vaccination status of LE patients differed significantly from that of DM patients (p=0.005) (**Figure 4**). 89.5% of DM patients were fully vaccinated compared to 77.1% of LE patients (p=0.010). 42.9% of the LE patients in our sample were Black compared to 7.5% of DM patients. In both the LE and DM patient samples, a higher proportion of white patients were fully vaccinated than Black patients (**Figure 5**). There was a trend towards increased vaccination rates in patients with DM, morphea, BP, Behcet’s disease, and MMP compared to other diseases such as LE and pemphigus (p=0.061) (**Figure 6**).

Of the patients who had autoimmune exacerbations after vaccination, 20% had flares after the first dose, 82% after the second dose, and 4% after the third dose. Some patients experienced disease flares after multiple doses. The median time to autoimmune exacerbation onset (95% confidence interval) was 7 (2, 14), 14 (14, 21), and 18 (7, 28) days after the first, second, and third vaccine doses, respectively.

## Discussion

Several studies have shown that some autoimmune disease patients experience flares after the COVID-19 vaccine (6–8). However, these studies used either on-line patient surveys or physician-reported registries to generate their data, representing possible limitations in determining true flare incidence rates. In this study, we prospectively and systematically evaluated patients to determine if they experienced autoimmune disease exacerbations following the COVID-19 vaccine. The rate of fully vaccinated patients reporting autoimmune disease exacerbations in our study (14.8%) is higher than the previously reported incidences of rheumatic disease flares (3%-13.4%) following COVID-19 inoculation (6–8). Perhaps this difference results from differences in the populations sampled in our study compared to past studies. Past studies that used online patient surveys and physician-reported registries are more likely to include patients with mild autoimmune disease than patients from our study which were all recruited from a single tertiary care center that specializes in autoimmune skin disease with a high volume of moderate-to-severe autoimmune disease patients. Since these sicker patients have increased flare rates generally, it is possible that they also have increased flare rates with specific environmental triggers, such as the COVID-19 vaccine. As our study included patients from a dermatology clinic, it is also possible that the ubiquitous prevalence of skin disease in our population played a role in the increased rate of disease exacerbations seen. As 19.6% of the fully vaccinated patients who experienced disease exacerbations in our study stopped their autoimmune disease treatment at the time of vaccination, it is possible that some of these flares were due to medication discontinuation. However, most patients who flared did not stop their medication for the vaccine and so it was not a factor contributing to their disease exacerbation.

The low rate of flares requiring an escalation in treatment in our study (6.7% of fully vaccinated patients or 45.1% of patients who flared) is reassuring and indicates that the COVID-19 vaccine is relatively safe and well tolerated by many patients with autoimmune skin disease. However, it does raise questions of risk-benefit analysis for patients who have had severe vaccine-related flares on the necessity of additional vaccine doses or booster shots. As new variant-specific vaccines are being developed, such as the Omicron-based vaccine, it is not clear either how safe these will be for autoimmune disease patients with past COVID-19 vaccine-related flares.

It is not totally obvious why the incidence of flares varied across diagnoses or specifically why DM patients were more likely to flare after the COVID-19 vaccine than LE patients. Only speculation is possible. Both DM and LE pathogenesis are believed to be in part driven by type 1 interferons which the COVID-19 vaccines have been shown to upregulate (11–13). However, the immune cells driving interferon expression in these diseases differ, with plasmacytoid dendritic cells playing a key role in LE interferon expression compared to myeloid dendritic cells in DM (14, 15). These differences in pathogenesis may be partly responsible for the differences in flares seen in these diseases after the COVID-19 vaccine. There is a lack of data comparing the flare rates of LE to DM patients, but studies looking at each disease individually report a lower rate of flares in LE than DM (16, 17). Thus, it is possible that more flares were seen in our study after the vaccine in DM patients than LE patients due to an increased tendency to flare at any given time.

Though the incidence in autoimmune disease exacerbation after vaccination did not differ significantly by COVID-19 vaccine manufacturer, there was a trend towards increased flares following the Moderna vaccine. Studies have shown the Moderna vaccine to be more immunogenic than the Pfizer-BioNTech or J&J vaccines, resulting in greater spike protein-specific antibody levels, neutralizing antibody levels, and spike protein-specific T cell responses (18). Data indicates that increased vaccine immunogenicity may correlate with increased vaccine-related side effects due to heightened immune system activation (18). This could explain why the more immunogenic Moderna vaccine had the highest rate of flares in our study as there was possibly an increased immune response with off-target autoimmune effects. There was also a trend towards the fewest autoimmune disease exacerbations occurring after the J&J vaccine in our study. This also seems to correlate with what is known about vaccine immunogenicity as J&J has been shown to result in lower levels of vaccine-induced antibodies and have a less robust T-cell response than the mRNA vaccines (18).

While the vaccination rate differed significantly between LE and DM patients with DM patients more likely to be fully vaccinated, this is likely explained in large part due to differences in the racial makeup of these two disease populations as well as variation in vaccine acceptance across racial backgrounds. COVID-19 vaccination rates among Black people in the US are lower than other racial groups (19). Black patients made up a larger share of LE patients in our study than DM patients and also had lower vaccination rates than white patients in both disease populations, which is consistent with previous data on differences in vaccination rates by race and helps to explain the differences observed across disease diagnoses.

Most patients who experienced autoimmune disease exacerbations after the COVID-19 vaccine in our study had increased symptoms after the second dose (82%). Studies have shown that higher levels of antibody response occur after the second dose of COVID-19 vaccines compared to the first dose (20). This increased humoral response may parallel the increased autoimmune response seen after the second COVID-19 vaccine dose in our study. Additionally, prior infection with COVID-19 has been shown to play a role in the level of immune response after each dose of the COVID-19 vaccine, with increased immunogenicity seen in previously infected individuals after the first vaccine dose (21). Thus, one possible mechanism for patients experiencing autoimmune disease exacerbations after the first vaccine dose is a prior COVID-19 infection which resulted in a more robust immune response after the first dose with increased autoimmune side effects.

There are several limitations to this study. Patients may have not accurately remembered details about their COVID-19 vaccine and any possible disease flares they experienced afterwards. Some patients were seen several months after they received the COVID-19 vaccine, increasing the possibility of recall bias. It is also possible that some patients who experienced flares temporally correlated with the COVID-19 vaccine were coincidental and that vaccine was not an autoimmune trigger. However, comparing the disease exacerbation rate by diagnosis and vaccine manufacturer helps to mitigate this potential limitation.

This study demonstrates that most patients with autoimmune skin disease tolerate the COVID-19 vaccine well, but a small number of patients do experience disease flares requiring an escalation in treatment (6.7%). Given the risks of hospitalization and death associated with COVID-19, clinicians should continue to promote vaccination in most patients.

## Data Availability Statement

The raw data supporting the conclusions of this article will be made available by the authors, without undue reservation.

## Ethics Statement

The studies involving human participants were reviewed and approved by Institutional Review Board at the University of Pennsylvania. Written informed consent to participate in this study was provided by the participants’ legal guardian/next of kin.

## Author Contributions

GS, EK, MG, JC, and VW conceived and designed this study. GS collected data and drafted the manuscript and figures. MA, JD, and RF performed the statistical analysis. VW supervised the work. All authors revised the article and approved the submitted version.

## Funding

This project is supported by the Department of Veterans Affairs Veterans Health Administration, Office of Research and Development, Biomedical Laboratory Research and Development, the National Institutes of Health (NIAMS) [R01AR076766].

## Conflict of Interest

The authors declare that the research was conducted in the absence of any commercial or financial relationships that could be construed as a potential conflict of interest.

## Publisher’s Note

All claims expressed in this article are solely those of the authors and do not necessarily represent those of their affiliated organizations, or those of the publisher, the editors and the reviewers. Any product that may be evaluated in this article, or claim that may be made by its manufacturer, is not guaranteed or endorsed by the publisher.

## References

[1] Islam S Islam T Islam MR. New Coronavirus Variants are Creating More Challenges to Global Healthcare System: A Brief Report on the Current Knowledge. Clin Pathol (2022) 15:2632010X221075584. doi: 10.1177/2632010X221075584 PMC8819824 35141522

[2] Lin D-Y Gu Y Wheeler B Young H Holloway S Sunny S-K. Effectiveness of Covid-19 Vaccines Over a 9-Month Period in North Carolina. N Engl J Med (2022) 386:933–41. doi: 10.1056/NEJMoa2117128 35020982 PMC8781317

[3] Tsai R Hervey J Hoffman K Wood J Johnson J Deighton D. COVID-19 Vaccine Hesitancy and Acceptance Among Individuals With Cancer, Autoimmune Diseases, or Other Serious Comorbid Conditions: Cross-Sectional, Internet-Based Survey. JMIR Public Health Surveill (2022) 8:e29872. doi: 10.2196/29872 34709184 PMC8734610

[4] Furer V Rondaan C Agmon-Levin N van Assen S Bijl M Kapetanovic MC. Point of View on the Vaccination Against COVID-19 in Patients With Autoimmune Inflammatory Rheumatic Diseases. RMD Open (2021) 7:e001594. doi: 10.1136/rmdopen-2021-001594 33627440 PMC7907831

[5] Schulze-Koops H Specker C Skapenko A. Vaccination of Patients With Inflammatory Rheumatic Diseases Against SARS-CoV-2: Considerations Before Widespread Availability of the Vaccines. RMD Open (2021) 7:e001553. doi: 10.1136/rmdopen-2020-001553 33627439 PMC7907835

[6] Machado PM Lawson-Tovey S Strangfeld A Mateus EF Hyrich KL Gossec L. Safety of Vaccination Against SARS-CoV-2 in People With Rheumatic and Musculoskeletal Diseases: Results From the EULAR Coronavirus Vaccine (COVAX) Physician-Reported Registry. Ann Rheum Dis (2021) 81:695–709. doi: 10.1136/annrheumdis-2021-221490 34972811

[7] Felten R Kawka L Dubois M Ugarte-Gil MF Fuentes-Silva Y Piga M. Tolerance of COVID-19 Vaccination in Patients With Systemic Lupus Erythematosus: The International VACOLUP Study. Lancet Rheumatol (2021) 3:e613–5. doi: 10.1016/S2665-9913(21)00221-6 PMC8294805 34312612

[8] Sattui SE Liew JW Kennedy K Sirotich E Putman M Moni TT. Early Experience of COVID-19 Vaccination in Adults With Systemic Rheumatic Diseases: Results From the COVID-19 Global Rheumatology Alliance Vaccine Survey. RMD Open (2021) 7:e001814. doi: 10.1136/rmdopen-2021-001814 34493645 PMC8424419

[9] Tomayko MM Damsky W Fathy R McMahon DE Turner N Valentin MN. Subepidermal Blistering Eruptions, Including Bullous Pemphigoid, Following COVID-19 Vaccination. J Allergy Clin Immunol (2021) 148(3):750–1. doi: 10.1016/j.jaci.2021.06.026 PMC8280592 34275656

[10] Gambichler T Hamdani N Budde H Sieme M Skrygan M Scholl L. Bullous Pemphigoid After SARS-CoV-2 Vaccination: Spike-Protein-Directed Immunofluorescence Confocal Microscopy and T-Cell-Receptor Studies. Br J Dermatol (2021) 186:728–31. doi: 10.1111/bjd.20890 PMC8653321 34773638

[11] Bengtsson AA Rönnblom L. Role of Interferons in SLE. Best Pract Res Clin Rheumatol (2017) 31:415–28. doi: 10.1016/j.berh.2017.10.003 29224681

[12] Wong D Kea B Pesich R Higgs BW Zhu W Brown P. Interferon and Biologic Signatures in Dermatomyositis Skin: Specificity and Heterogeneity Across Diseases. PLos One (2012) 7:e29161. doi: 10.1371/journal.pone.0029161 22235269 PMC3250414

[13] Sadarangani M Marchant A Kollmann TR. Immunological Mechanisms of Vaccine-Induced Protection Against COVID-19 in Humans. Nat Rev Immunol (2021) 21:475–84. doi: 10.1038/s41577-021-00578-z PMC8246128 34211186

[14] Little AJ Vesely MD. Cutaneous Lupus Erythematosus: Current and Future Pathogenesis-Directed Therapies. Yale J Biol Med (2020) 93:81–95. 32226339 PMC7087060

[15] Chen KL Patel J Zeidi M Wysocka M Bashir MM Patel B. Myeloid Dendritic Cells Are Major Producers of IFN-β in Dermatomyositis and May Contribute to Hydroxychloroquine Refractoriness. J Invest Dermatol (2021) 141:1906–914.e2. doi: 10.1016/j.jid.2020.12.032 33675790 PMC8316264

[16] Christopher-Stine L Wan GJ Kelly W McGowan M Bostic R Reed ML. Patient-Reported Dermatomyositis and Polymyositis Flare Symptoms are Associated With Disability, Productivity Loss, and Health Care Resource Use. J Manag Care Spec Pharm (2020) 26(11):1424–33. doi: 10.18553/jmcp.2020.26.11.1424 PMC10391285 33119444

[17] Conti F Ceccarelli F Perricone C Miranda F Truglia S Massaro L. Flare, Persistently Active Disease, and Serologically Active Clinically Quiescent Disease in Systemic Lupus Erythematosus: A 2-Year Follow-Up Study. PLos One (2012) 7(9):e45934. doi: 10.1371/journal.pone.0045934 23029327 PMC3448702

[18] Sablerolles RSG Rietdijk WJR Goorhuis A Postma DF Visser LG Geers D. Immunogenicity and Reactogenicity of Vaccine Boosters After Ad26.COV2.S Priming. N Engl J Med (2022) 386:951–63. doi: 10.1056/NEJMoa2116747 35045226 PMC8796791

[19] Padamsee TJ Bond RM Dixon GN Hovick SR Na K Nisbet EC. Changes in COVID-19 Vaccine Hesitancy Among Black and White Individuals in the US. JAMA Netw Open (2022) 5:e2144470. doi: 10.1001/jamanetworkopen.2021.44470 35061038 PMC8783270

[20] Wheeler SE Shurin GV Yost M Anderson A Pinto L Wells A. Differential Antibody Response to mRNA COVID-19 Vaccines in Healthy Subjects. Microbiol Spectr (2021) 9:e0034121. doi: 10.1128/Spectrum.00341-21 34346750 PMC8552678

[21] Hirotsu Y Amemiya K Sugiura H Shinohara M Takatori M Mochizuki H. Robust Antibody Responses to the BNT162b2 mRNA Vaccine Occur Within a Week After the First Dose in Previously Infected Individuals and After the Second Dose in Uninfected Individuals. Front Immunol (2021) 12:722766. doi: 10.3389/fimmu.2021.722766 34512649 PMC8427169

